# *In vivo* Probabilistic Structural Atlas of the Inferior and Superior Colliculi, Medial and Lateral Geniculate Nuclei and Superior Olivary Complex in Humans Based on 7 Tesla MRI

**DOI:** 10.3389/fnins.2019.00764

**Published:** 2019-08-07

**Authors:** María G. García-Gomar, Christian Strong, Nicola Toschi, Kavita Singh, Bruce R. Rosen, Lawrence L. Wald, Marta Bianciardi

**Affiliations:** ^1^Department of Radiology, Athinoula A. Martinos Center for Biomedical Imaging, MGH and Harvard Medical School, Boston, MA, United States; ^2^Department of Neurosurgery, Brigham and Women’s Hospital, Harvard Medical School, Boston, MA, United States; ^3^Medical Physics Section, Department of Biomedicine and Prevention, Faculty of Medicine, Tor Vergata University of Rome, Rome, Italy

**Keywords:** inferior/superior colliculi, medial/lateral geniculate nuclei, superior olivary complex, *in vivo* neuroimaging-based human atlas, multi-contrast 7 Tesla MRI, visual/oculo-motor and auditory/auditory-motor functions

## Abstract

Despite extensive neuroimaging research of primary sensory cortices involved in auditory and visual functions, subcortical structures within these domains, such as the inferior and superior colliculi, the medial and lateral geniculate nuclei and the superior olivary complex, are currently understudied with magnetic resonance imaging (MRI) in living humans. This is because a precise localization of these nuclei is hampered by the limited contrast and sensitivity of conventional neuroimaging methods for deep brain nuclei. In this work, we used 7 Tesla multi-modal (T_2_-weighted and diffusion fractional anisotropy) 1.1 mm isotropic resolution MRI to achieve high sensitivity and contrast for single-subject brainstem and thalamic nuclei delineation. After precise coregistration to stereotactic space, we generated an *in vivo* human probabilistic atlas of auditory (medial geniculate nucleus, inferior colliculus, and superior olivary complex) and visual (lateral geniculate nucleus and superior colliculus) subcortical nuclei. We foresee the use of this atlas as a tool to precisely identify the location and shape of auditory/visual deep nuclei in research as well as clinical human studies.

## Introduction

Brainstem nuclei such as the inferior colliculus (IC), superior colliculus (SC) and the superior olivary complex (SOC), as well as thalamic nuclei such as the medial geniculate nucleus (MG) and lateral geniculate nucleus (LG) modulate auditory/auditory-motor (IC, SOC and MG) and visual/oculo-motor (SC and LG) functions. These nuclei are also involved in the pathogenesis of disorders such as auditory agnosia, pure-word deafness, eye-movement and visual-field deficits, hallucinations in Parkinson’s disease, and glaucoma (Joswig et al., 2015; Pasu et al., 2015; Wang et al., 2015; Biotti et al., 2016; Lee et al., 2016). Nevertheless, a stereotaxic probabilistic structural atlas of these nuclei in living humans does not exist. This limits research and clinical (diagnostic, prognostic, pharmaceutical, surgical) studies of their structure, function and connectivity in health and disease.

We briefly review the localization and the anatomical connectivity of the aforementioned structures. The IC, an oval shaped region located in the caudal half of the mesencephalic tectum, receives inputs from several brainstem auditory pathway nuclei such as the cochlear nuclei, nuclei of the lateral lemniscus, lateral and medial superior olive as well as from the auditory cortex. One of the efferent pathways from IC projects to the deeper layers of the SC (thus coordinating eye and head orientation in response to visual/auditory stimuli) while another ascends in the brachium of the IC targeting the MG in the thalamus (Olszewski and Baxter, 2014). The SC is a gray matter nucleus located in the rostral half of the mesencephalic tectum. Its primary function is the orientation of the head toward objects of interest. The optical tract is a major afferent of this nucleus. The SC also receives projections from the parabigeminal nucleus, substantia nigra pars reticulata, zona incerta and corticotectal projections from visual cortical areas and the medial temporal cortex (Olszewski and Baxter, 2014; May, 2006). The SC plays a key role for generation of saccadic eye movements, as it projects to the paramedian pontine reticular formation through the descending predorsal bundle; meanwhile its ascending branch projects toward the interstitial nucleus of Cajal, a vertical saccade generator in the midbrain, and to the mediodorsal thalamic nucleus and the intralaminar thalamic nuclei. The major output of the SC projects toward the LG in its way to the visual cortex (Olszewski and Baxter, 2014; May, 2006). The SOC contains two principal nuclei: the medial superior olivary nucleus and the lateral superior olivary nucleus. The SOC is located in the ventrolateral border of the caudal pontine tegmentum. The function of this nucleus is to process the binaural input converging from the cochlear nuclei from the two ears. The SOC projects to the IC via the lateral lemniscus (Kulesza, 2007). The MG is the thalamic relay of the auditory pathway. It is located in the ventromedial thalamus, it receives input from the IC and projects toward the auditory cortex. Finally, the LG is the thalamic relay of the visual pathway and is located in the ventrolateral thalamus. The LG receives input form the SC and projects toward the visual cortex.

As opposed to previous work performing indirect localization of the MG and LG based on fMRI and DTI connectivity analyses (Chen et al., 1999; Bestmann et al., 2004; Kastner et al., 2004; Schneider et al., 2004; Barnes et al., 2010; Javad et al., 2014; Keifer et al., 2015), we aimed at directly localizing these and the other aforementioned nuclei from *in vivo* structural magnetic resonance images (MRI). Previous structural MRI work reports the automatic/semi-automatic segmentation of some thalamic nuclei at 1.5 Tesla with 3 mm resolution (Wiegell et al., 2003), including the MG and LG. Additionally, previous studies performed automatic segmentation of the LG in an attempt to compare human healthy controls to glaucoma patients using T_1_-weighted images acquired with 3 Tesla and 1.5 Tesla MRI with 1.30 and 1.20 mm resolution respectively (Wang et al., 2015), as well as semi-automated segmentation of the LG to characterize its morphometry at 3 Tesla and 1.5 Tesla MRI with a voxel size of 1 mm (Hernowo et al., 2011; Cecchetti et al., 2016). Nevertheless, these studies mainly focused on single-subject nuclei segmentations rather than creating *in vivo* human probabilistic (i.e., representative of a population) atlases of the visual and auditory thalamic/brainstem nuclei.

Both auditory and visual pathways demonstrated lower gray matter volumes in disease states relative to healthy controls (specifically, lower volume of the right IC and left hippocampus in tinnitus patients (Landgrebe et al., 2009) or lower LG volume in blind patients relative to sighted individuals (Aguirre et al., 2016)). Similarly, other studies suggest that visual deficits can be predicted by delineation of the optic tract (de Blank et al., 2018), while other studies report being able to track the optic neuritis recovery by measuring functional activation in the LG (Korsholm et al., 2007). An improved understanding of the anatomical boundaries of these nuclei may better elucidate such prognostic frameworks. Further, acoustic pathway delineation is limited by current MRI techniques (Maffei et al., 2018). In this context, an improved localization of auditory nuclei such as the MG, IC and SOC might help better characterize this pathway. Finally, LG is relevant in psychiatric diseases like schizophrenia (Mai et al., 1993; Selemon and Begovic, 2007; Dorph-Petersen et al., 2009; Buchmann et al., 2014).

The primary objective of our work was to create an *in vivo* probabilistic neuroimaging-based atlas of the right and left IC, SC, MG, LG and SOC using: (1) cutting-edge technology (7 Tesla MRI scanner, 32-channel receive coil-array) to maximize MRI detection sensitivity; (2) a high-resolution (1.1 mm isotropic) multi-contrast [T_2_-weighted and diffusion fractional anisotropy (FA)] echo-planar-imaging (EPI) approach, which provided complementary contrasts for brainstem anatomy with precisely matched geometric distortions and resolution.

## Materials and Methods

### MRI Data Acquisition

Data were obtained in a prior study (Bianciardi et al., 2015). While the acquisition and analysis are summarized below, full details can be found in Bianciardi et al., 2015. Twelve healthy subjects (6m/6f, age 28 ± 1 years) provided informed and written consent for 7 Tesla MRI (Magnetom, Siemens Healthineers, Germany) per Massachusetts General Hospital Institutional Review Board approval in accordance with the Declaration of Helsinki. A custom-built 32-channel receive coil and volume transmit coil was employed in data acquisition (Keil et al., 2010), providing improved sensitivity for the brainstem compared to commercial coils with a similar number of channels. The improved sensitivity of this coil was due to: (i) a more posterior arrangement of the coil elements; (ii) its shape curving around the back of the head enabling a closer proximity to the cerebellum and brainstem as opposed to commercial coils that typically extend straight down along the lower parts of the head; (iii) a more efficient flip angle calibration for lower parts of the brain because the coil extends more inferiorly than traditional coils. We utilized a common single-shot 2D echo-planar imaging scheme for 1.1 mm isotropic sagittal diffusion-tensor images (DTI), and T_2_-weighted images, with matrix size/GRAPPA factor/nominal echo-spacing = 180 × 240/3/0.82 ms. This provided T_2_-weighted anatomical images with resolution and geometric distortions perfectly matched to the DTI dataset. With the echo-planar-imaging scheme, we were also able to mitigate specific-absorption-rate limits of spin-warp T_2_-weighted MRI at 7 Tesla. Other specifications of DTI and T_2_-weighted images included: spin-echo EPI, 61 slices, echo-time/repetition-time = 60.8 ms/5.6 s, partial Fourier: 6/8, unipolar diffusion-weighting gradients (for DTI), 60 diffusion directions (for DTI, *b*-value ∼ 1000 s/mm^2^), 7 interspersed “b0” images (non-diffusion weighted, *b*-value ∼ 0 s/mm^2^, which were also utilized as T_2_-weighted MRI), 4 repetitions, acquisition time/repetition 6’43”. The total acquisition time for DTI and T_2_-weighted MRI was ∼27’. Importantly, use of unipolar (Stejskal and Tanner, 1965) rather than bipolar (Reese et al., 2003) diffusion gradients allowed shortening of the echo-time by ∼30 ms and hence provided significantly improved sensitivity of high-resolution DTI.

### MRI Data Pre-processing and Alignment to MNI Space

On an individual subject basis, the diffusion FA map was calculated from the DTI acquisition concatenated across 4 repetitions and preprocessed (distortion and motion-corrected) with the Diffusion Toolbox of the FMRIB Software Library (FSL, Oxford, United Kingdom) as in Bianciardi et al. (2015). After motion correction, the 28 “b0” T_2_-weighted images were averaged and co-registered to DTI data via affine transformation.

Co-registration to MNI space was performed on a single-subject basis for both FA and T_2_-weighted images as in Bianciardi et al. (2015). Each subject’s FA image was aligned to an MNI space-based diffusion FA template (coined “IIT space”) (IIT human brain atlas, v.3, Chicago, IL, United States) (Varentsova et al., 2014). This template was utilized because it encompasses the entirety of the brainstem, is compatible with diffusion-based tractography, and provides high contrast detail in the brainstem. Specifically, we utilized the Advanced Normalization Tool (ANTs, Philadelphia, PA, United States) (Avants et al., 2011) by concatenating a generic affine and a high-dimensional non-linear warp transformation computed for images having the same modality (FA maps). The generic affine transformation was computed by concatenating center-of mass alignment (degrees of freedom, dof = 3), rigid (dof = 6), similarity (dof = 7) and fully affine (dof = 12) transformations with smoothing sigmas: 4, 2, 1, 0 voxels – fixed image space. The high-dimensional non-linear warp transformation employed a symmetric diffeomorphic normalization transformation model with smoothing sigmas: 3, 2, 1, 0 voxels – fixed image space –, and histogram image matching prior to registration. It also employed a cross correlation metric, regular sampling, gradient step size: 0.2; four multi-resolution levels: shrink factors 6, 4, 2, 1 voxels – fixed image space; data winsorization – quantiles: 0.005, 0.995; convergence criterion: slope of the normalized energy profile over the last 10 iterations <10^-8^. The combined transformation was then applied to both single-subject FA and T_2_-weighted images, using a single-interpolation step (interpolation method: linear). Single-subject FA and T_2_-weighted images were also aligned to MNI152 standard (non-linear 6th generation MNI152_T1_1mm available for instance in FSL) space (coined “MNI152_1mm space”), which is frequently utilized space for fMRI analysis. While the MNI152_1mm space and the IIT space are well aligned elsewhere, there is slight misalignment in the brainstem, particularly in the pons and medulla. Therefore, single-subject FA and T_2_-weighted images were aligned to MNI152_1mm space by applying two concatenated transformations, using a single-interpolation step (interpolation method: linear): first, the single-subject to IIT space transformation computed above; and second, the IIT to MNI152_1mm non-linear transformation computed in Bianciardi et al. (2015), using the same parameters as above.

### Single-Subject Labeling and Probabilistic Atlas Generation

On a single-subject basis, two raters (C.S. and M.B. for IC, SC, and MG; M.G. and M.B for LG and SOC) independently performed a manual segmentation (fslview, FSL, Oxford, United Kingdom) of multi-contrast (FA maps and T_2_-weighted) images in IIT space to yield single-subject labels (i.e., masks) of the regions of interest (ICl/r, SCl/r, MGl/r, LGl/r, SOCl/r). Each rater inspected both imaging modalities simultaneously. For each nucleus in each subject, we defined a “*final label”* including only voxels marked by both raters (i.e., the intersection of the labels of the two raters). The manual segmentation was performed by utilizing image contrast and anatomical landmarks (Paxinos et al., 2012) as follows.

The IC was delineated based on a hypointense region in T_2_-weighted images located in the tectum of the midbrain, caudal to the SC on the dorsal aspect of the mesencephalon, lateral –in its dorsal aspect- to the periaqueductal gray (hyperintense in T_2_-weighted images), and –ventrally– to the cuneiform nucleus (hypointense in the FA map). The IC was bounded laterally by the cerebrospinal fluid. The SC was visible in the midbrain as a hypointense region on T_2_-weighted images, rostral to the IC, lateral to the periaqueductal gray and bounded laterally by the cerebrospinal fluid. The MG was identified as a small rounded/oval shaped eminence hypointense in T_2_-weighted images, adjacent to the corticospinal tract (hyperintense in the FA map), pulvinar nucleus (hypointense in T_2_-weighted images), LG (hypointense in FA maps), and substantia nigra (hypointense in T_2_-weighted images). The LG was identified as a “rainbow-shaped” ovoid structure hypointense in FA maps in the posteroinferior thalamus, located lateral to the corticospinal tract (hyperintense in the FA map), and anterolateral to the MG (hypointense in T_2_-weighted images). The SOC, comprising the lateral and medial superior olive, the lateroventral and medioventral periolivary nucleus, and the superior paraolivary nucleus, was identified on FA maps as a hypointense oval region dorsolateral to the central tegmental tract, and with its most caudal part posterior to the superior border of the inferior olive.

Note that, to delineate each nucleus, we mainly used the image modality (either FA or T_2_-weighted MRI – for instance T_2_-weighted MRI for IC, SC and MGN; FA for LG and SOC) that displayed the nucleus boundaries with good contrast, and employed the other modality (which had poor contrast for that nucleus – e.g., FA for IC, SC and MGN; T_2_-weighted MRI for LG and SOC) to identify neighboring nuclei or landmarks.

A probabilistic neuroimaging atlas in IIT space was formulated for each nucleus as an average probability map of the nucleus label encompassing all subjects (highest probability = 100% overlap of nuclei labels across subjects, *n* = 12). After registering the individual subject labels to MNI152_1mm space (by applying the IIT to MNI152 transformations described above, interpolation method: nearest neighbor in order to preserve label intensities), a probabilistic neuroimaging atlas in MNI152_1mm spaces of these nuclei was also formulated. The atlas was developed in both IIT and MNI152 spaces to facilitate extrapolation to structural, diffusion and functional MRI modalities.

For each subject and each final label (coregistered to single-subject native space via the inverse of the transformations described in 2.2) we also calculated the label volume in native space. We then computed the mean (s.e.) label volume across all subjects.

### Atlas Validation

The probabilistic nuclei atlas was validated by computing for each nucleus and subject: (i) the inter-rater agreement, as the modified Hausdorff distance between labels delineated by the two raters; (ii) the internal consistency across subjects of the final label, as the modified Hausdorff distance between each final label and the probabilistic atlas label (thresholded at 35%) generated by averaging the labels across the other 11 subjects (leave-one-out cross validation). For both (i) and (ii), the modified Hausdorff distance (Dubuisson and Jain, 1994), a measure of spatial overlap frequently used in neuroimaging (Fischl et al., 2008; Ghosh et al., 2010; Klein et al., 2010; Yendiki et al., 2011; Augustinack et al., 2013), was calculated as follows: the minimum distance of each point within one label from the other label was averaged across all points for each label, resulting in two distance values; the maximum value of these 2 values was calculated. We also evaluated the inter-rater agreement and the internal consistency of labels with a metric more commonly used in the literature (especially for larger brain structures), the Dice similarity coefficient (Dice, 1945). The Dice similarity coefficient of two labels X and Y (e.g., of the two raters for the inter-rater agreement) is defined as (2^∗^|*X*∩*Y*|)/(|*X*| + |*Y*|), where |*X*| + |*Y*| are the volumes of each label and |*X*∩*Y*| is the volume of the union of the two labels. Finally, for each nucleus, the modified Hausdorff distance as well as the Dice similarity coefficient in both cases (i) and (ii) was averaged across subjects.

## Results

**FIGURE 1:**
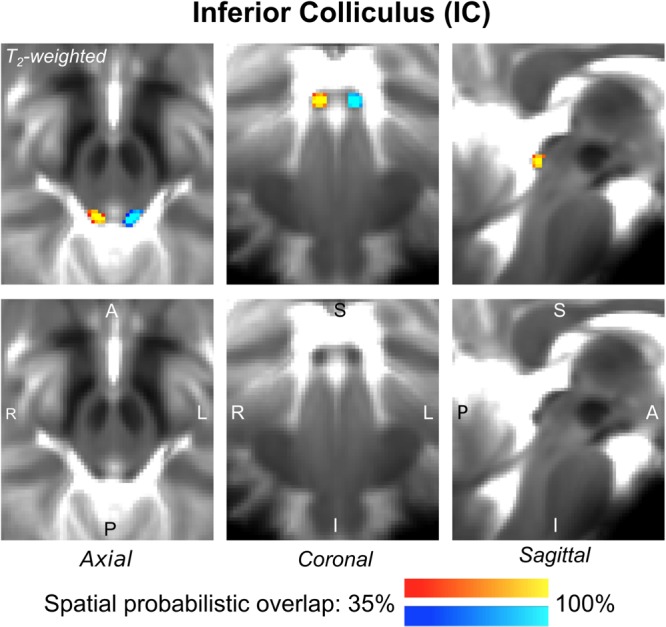
Probabilistic atlas label in MNI space of the IC (**left:** blue-to-cyan; **right:** red-to-yellow). The IC was hypointense in T_2_-weighted MRI; its label is overlaid on the group average T_2_-weighted image. Very good (i.e., up to 100%) spatial agreement of labels across subjects was observed indicating the feasibility of delineating the probabilistic label of this nucleus involved in auditory and auditory-motor functions.

**FIGURE 2:**
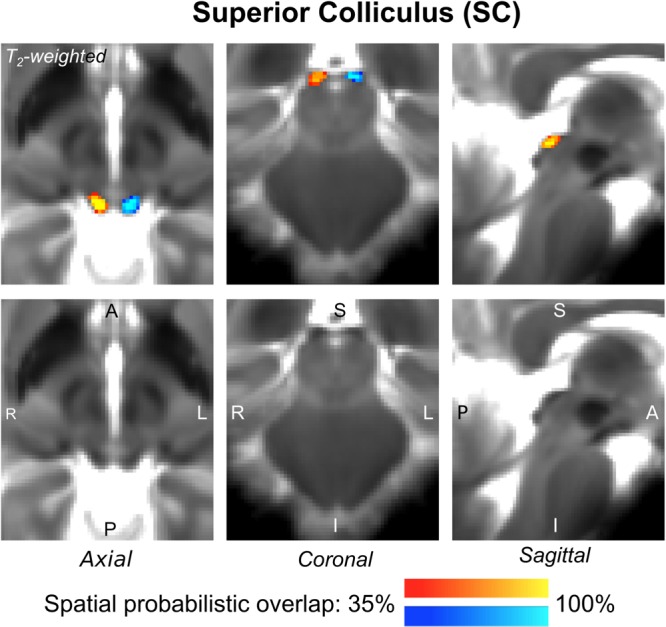
Probabilistic atlas label in MNI space of the SC (**left:** blue-to-cyan; **right:** red-to-yellow). The SC was hypointense in T_2_-weighted MRI; its label is overlaid on the group average T_2_-weighted image. Very good (i.e., up to 100%) spatial agreement of labels across subjects was observed indicating the feasibility of delineating the probabilistic label of this nucleus involved in visual and oculo-motor functions.

**FIGURE 3:**
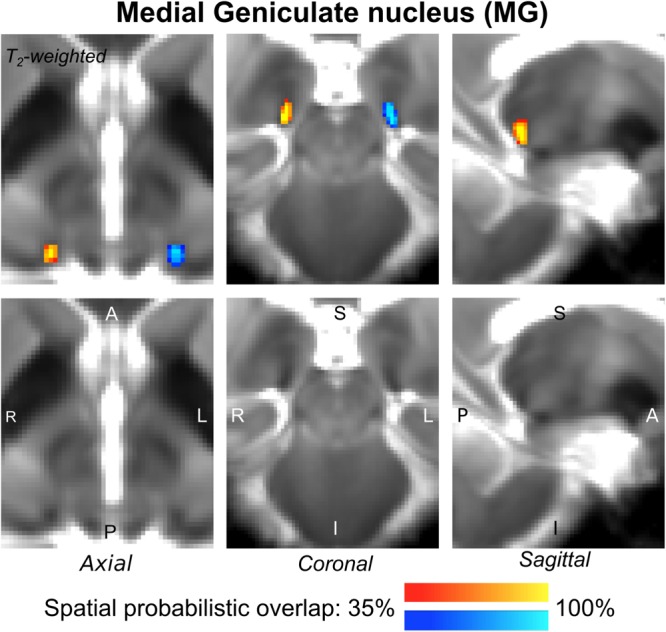
Probabilistic atlas label in MNI space of the MG (**left:** blue-to-cyan; **right:** red-to-yellow). The MG was hypointense in T_2_-weighted MRI; its label is overlaid on the group average T_2_-weighted image. Very good (i.e., up to 100%) spatial agreement of labels across subjects was observed indicating the feasibility of delineating the probabilistic label of this nucleus, which is a thalamic relay between the IC and the auditory cortex, and thus it is crucially involved in auditory and auditory-motor functions.

**FIGURE 4:**
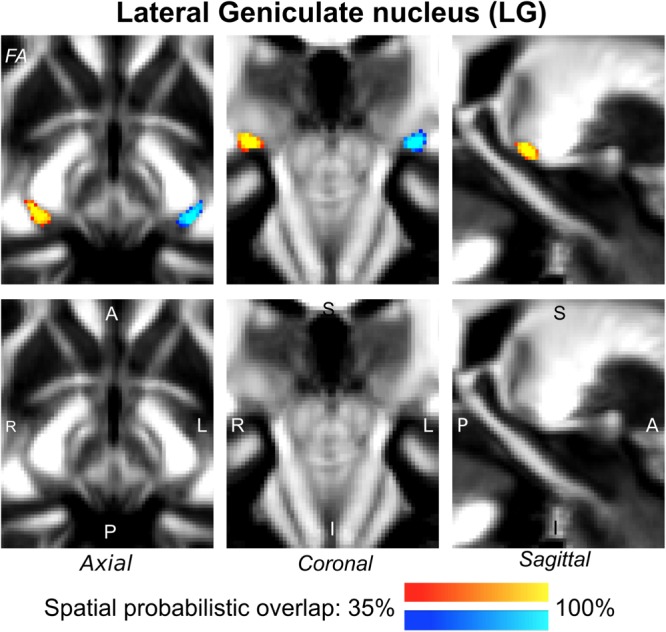
Probabilistic atlas label in MNI space of the LG (**left:** blue-to-cyan; **right:** red-to-yellow). The LG was hypointense in diffusion FA MRI; its label is overlaid on the group average FA map. Very good spatial (i.e., up to 100%) agreement of labels across subjects was observed indicating the feasibility of delineating the probabilistic label of this nucleus, which is a thalamic relay for visual and oculo-motor pathways.

**FIGURE 5:**
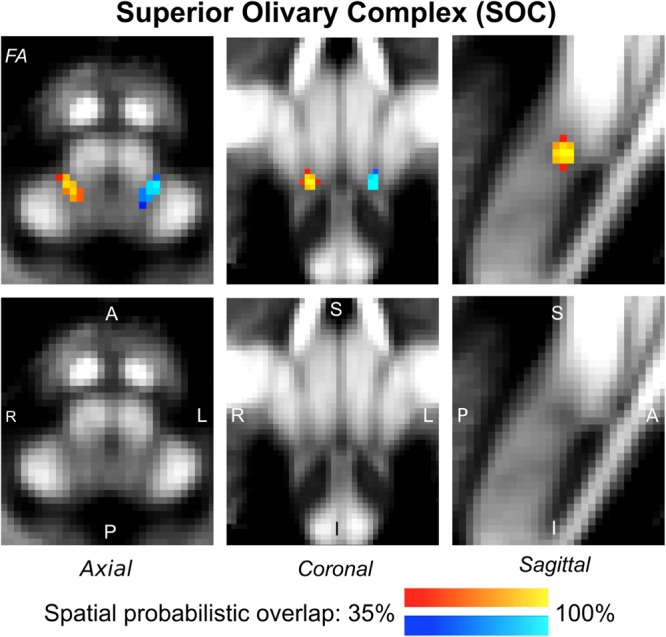
Probabilistic atlas label in MNI space of the SOC (**left:** blue-to-cyan; **right:** red-to-yellow). The SOC was hypointense in diffusion FA MRI; its label is overlaid on the group average FA map. Very good spatial (i.e., up to 100%) agreement of labels across subjects was observed indicating the feasibility of delineating the probabilistic label of this nucleus, which is a brainstem structure involved in auditory and auditory-motor functions.

The probabilistic neuroimaging-based structural labels in MNI space of ICl/r are shown in Figure 1. The left and right IC nuclei appeared as hypointensities compared to neighboring regions on T_2_-weighted MRI. The probabilistic neuroimaging-based structural labels in MNI space of SCl/r are shown in Figure 2. Again, the left and right nuclei appeared as hypointensities on T_2_-weighted MRI, possibly indicating a higher iron concentration compared to neighboring areas (Drayer et al., 1986). In Figure 3, we show the probabilistic atlas label in MNI space of the MGl/r. The MG was also hypointense in T_2_-weighted MRI. In Figure 4, we show the probabilistic atlas label in MNI space of the LGl/r, which was hypointense in the FA map. In Figure 5, we show the probabilistic atlas label in MNI space of the SOCl/r, which was an oval hypointense structure in the FA map. All the nuclei (ICl/r, SCl/r, MGl/r, LGl/r and SOCl/r) demonstrated good spatial (i.e., up to 100%) agreement of labels across subjects indicating the feasibility of delineating the probabilistic label of these nuclei in standard space.

**FIGURE 6:**
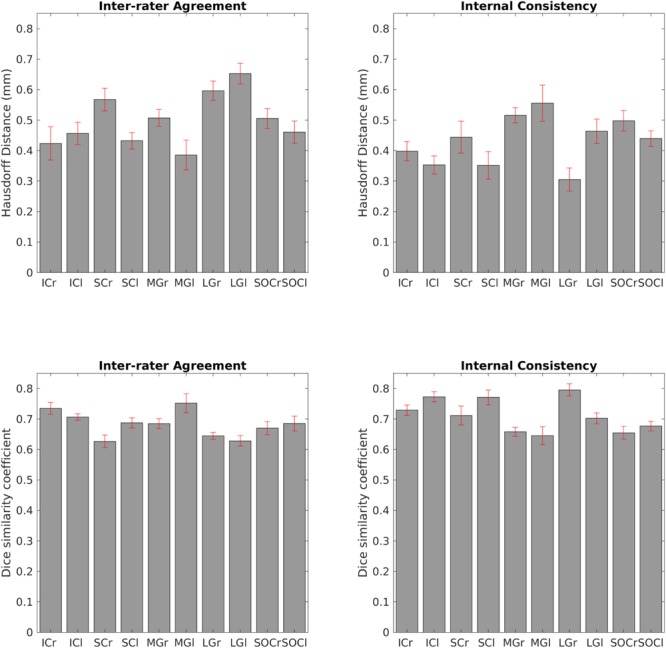
Atlas validation. **Upper left:** inter-rater agreement of nuclei labels (bar/error bar = mean/s.e. modified Hausdorff distance across 12 subjects). **Upper right:** internal consistency of nuclei labels across subjects (bar/errorbar = mean/s.e. modified Hausdorff distance across 12 subjects). **Lower left:** Dice similarity coefficient (range: 0–1) of the inter-rater agreement (bar/error bar = mean/s.e. Dice similarity coefficient across 12 subjects) **Lower right:** Dice similarity coefficient (range: 0–1) of the internal consistency (bar/error bar = mean/s.e. Dice similarity coefficient across 12 subjects). The labels of the IC, SC, MG, LG and SOC displayed good spatial overlap across raters and subjects (i.e., modified Hausdorff distance smaller than the imaging resolution and Dice similarity coefficient higher than 0.63), thus validating the probabilistic nuclei atlas.

The inter-rater label agreement and the internal consistency of each label (computed for validation) are both shown in Figure 6. For each nucleus, the average modified Hausdorff distance assessing the inter-rater agreement and the internal consistency of nuclei atlas labels (Figure 6, upper row) was below the linear spatial imaging resolution (1.1 mm) (*p* < 0.05, unpaired *t*-test), thus confirming the accuracy of the delineations of two independent raters. In addition, the Dice similarity coefficient for the inter-rater agreement and the internal consistency (Figure 6, bottom row) was always above 0.63, thus showing a good performance of both metrics.

In Table 1, for each nucleus we report the mean (± s.e.) volume (as well as volume range) across 12 subjects obtained in the present study. Furthermore, in Table 1 we also report an extensive literature review of volumes of IC, SC, MG, LG and SOC obtained in postmortem histological studies, as well as in structural and functional MRI. We mostly included studies performed in younger human adults [age between 19 and 47 years, except for two studies on older subjects (Nara et al., 1996; Sitek et al., 2019)] because it is known that the brain displays morphological changes with aging (Keuken et al., 2013). Except for the SC, the nuclei volumes obtained in this study fell (*p* < 0.05, *t*-test) within the range of literature values.

**Table 1:** Volumes of the IC, SC, MG, LG, and SOC.

NUCLEUS name (acronym)	Prior studies										Current study	

	Histology			Structural MRI			fMRI					
	Volume (mm^3^) (range)	Age (years)	References	Volume (mm^∗^) (range)	Age (years) (range or mean ± s.e)	References	Volume (mm^∗^) (range)	Age (years) (range or mean ± s.e)	References	Cross-modal^∗^ Volume range (mm^3^)	Right nucleus Volume (mm^3^) mean ± s.e (range)	Left nucleus Volume (mm^3^) mean ± s.e. (range)
Inferior Colliculus (IC)	63	65	Sitek et al., 2019	73	65	Sitek et al., 2019	200	25–30	Sitek et al., 2019	20.7–200	60 ± 3 (40–77)	55 ± 3 (41–69)
	20.7	63	Nara et al., 1996	119–165	28	Bestmann et al., 2004	193–194	20	Amaral et al., 2016			
	65. 2	–	Glendenning and Masterton, 1998				111–118	32 ± 9	Cecchetti et al., 2016			
							124–135	27 ± 6	Kang et al., 2008			
Superior Colliculus (SC)	–	–	–	90–110	20–35	Schneider and Kastner, 2009	171–176	20	Amaral et al., 2016	86–180	71 ± 5 (44–95)	75 ± 4 (44–95)
				86	22–35	Schneider and Kastner, 2005	170–190	32 ± 9	Cecchetti et al., 2016			
							158- 165	27 ± 6	Kang et al., 2008			
Medial Geniculate Nucleus (MGN)	75	65	Sitek et al., 2019	134	65	Sitek et al., 2019	285	25–30	Sitek et al., 2019	46.6–285	57 ± 6 (28–101)	66 ± 8 (35–126)
	58	–	Glendenning and Masterton, 1998	115–116	*20*	Amaral et al., 2016	185	28	Bestmann et al., 2004			
	46.6–71.2	28, 32	Armstrong, 1979	129	37 ± 7	Kitajima et al., 2015						
	105	–	Hopf, 1965									
Lateral Geniculate Nucleus (LGN)	104	–	Dorph-Petersen et al., 2009	87.7–88.8	47 ± 9	Schmidt et al., 2018	579	30 ± 4	Barnes et al., 2010	66–579	101 ± 5 (83–126)	93 ± 6 (73–129)
	66–152	–	Zvorykin, 1981	133–135	32 ± 9	Cecchetti et al., 2016	121–129	20–35	Schneider and Kastner, 2009			
	416	–	Stephan et al., 1981	117–144	37 ± 12	Wang et al., 2016	215	22–35	Schneider and Kastner, 2005			
	91–157	32	Andrews et al., 1997	250–274	20	Amaral et al., 2016	234–244	21–38	Kastner et al., 2004			
	69–78	28, 32	Armstrong, 1979	135	37 ± 7	Kitajima et al., 2015	440	22–30	Schneider et al., 2004			
	77–115	–	Putnam, 1926	70–170	51 ± 15	Wang et al., 2015						
				112–120	22–26	Giraldo-Chica et al., 2015	239–258	22–38	O’Connor et al., 2002			
				159	30 ± 12	Kelly et al., 2014	270	19–29	Chen et al., 1999			
				110	37	Hernowo et al., 2014						
				93–106	45 ± 11	Lee et al., 2014						
				90–177	35	Chen et al., 2013						
				158–165	32 ± 3	Mcketton et al., 2014						
				52–105	38 ± 14	Li et al., 2012						
				129.6–143.5	35	Dai et al., 2011						
				267	32	Korsholm et al., 2007						
				270	20–37	Horton et al., 1990						
Superior Olivary Complex (SOC)	6	65	Sitek et al., 2019	4	65	Sitek et al., 2019	124	25–30	Sitek et al., 2019	4–124	16 ± 1 (11–21)	20 ± 1 (12–27)
	5.76	54–76	Hilbig et al., 2009^†^									
	5.82	–	Glendenning and Masterton, 1998^†^									

*^∗^Cross-modal across prior histological, structural MRI and fMRI studies.*

*^†^The reported values only include the volume of the lateral and medial olivary complex.*

## Discussion

Compared to a large body of research focused on primary auditory/visual cortical areas (Ress et al., 2000; Morosan et al., 2001; Rademacher et al., 2001; Abdul-Kareem and Sluming, 2008; Hinds et al., 2009; Hoffmann et al., 2009; Bridge, 2011; Sánchez-Panchuelo et al., 2012; Augustinack et al., 2014; Saenz and Langers, 2014; Wasserthal et al., 2014; Cheng, 2016; Moerel et al., 2019), the structure, function and connectivity of subcortical brainstem and thalamic auditory/visual nuclei are currently understudied. New tools are needed to fill this gap and enable accurate structural delineations of the underlying deep brain nuclei in future clinical and research studies.

We first review the technological and methodological advances of this study that overcame conventional imaging limitations and enabled the generation of a new tool, namely a structural probabilistic atlas of subcortical auditory/visual nuclei. Then, for each nucleus, we discuss the segmentation process, in relation to the MRI contrast used for the nucleus delineation, the identification of specific nuclei borders, and the obtained volumes in comparison to literature values. Further, we discuss the possible impact of the atlas for clinical and research studies. Finally, we acknowledge the limitations of this work and propose possible future extensions.

### Technological and Methodological Advances

In this work we showed that the use of advanced ultra-high-field MRI sequences allowed *in vivo* structural imaging of tiny brainstem and thalamic nuclei with high contrast, sensitivity and good spatial resolution with respect to conventional (e.g., 3 Tesla) MRI. For instance, a 7 Tesla diffusion MRI is expected to yield a ∼2.2 increase in sensitivity with respect to 3 Tesla MRI, and gains in contrast related to decreased partial volume effects due to increased spatial resolution. Notably, the 7 Tesla sequence was optimized to: (i) achieve minimum echo time (e.g., by the use of a monopolar scheme) given the lower gray and white matter T_2_ values at 7 Tesla compared to 3 Tesla; (ii) refine the RF transmit gain in the brainstem (to compensate for strong RF in-homogeneities at 7 Tesla), by tuning the RF voltage with an actual flip-angle imaging pulse sequence (Yarnykh, 2007); (iii) perform several iterations of semi-automatic B_0_ shimming. Specifically, these 7 Tesla structural MRI (T_2_-weighted and FA maps) techniques enabled the single-subject segmentation of subcortical auditory and visual subcortical nuclei based on their contrast with respect to neighboring areas. An important consideration regards the multi-contrast, resolution- and distortion-matched structural image acquisition used in this work. The latter allowed us to discriminate the structural boundaries of some nuclei due to the improved visualization of multiple contrasts in a common space, and to the use of complementary information derived from different image contrasts.

### On the Atlas Creation

Our findings demonstrate the feasibility of delineating auditory/visual brainstem and thalamic nuclei by segmentation of single-subject high-contrast and high-sensitivity MRI images at 7 Tesla. This extends previous reports (Li et al., 2012) of manual single-subject localization of LG using neuroimaging in living humans based on the identification of anatomical landmarks. Crucially, our work also demonstrated the feasibility of generating a validated *in vivo* stereotaxic probabilistic atlas of these structures after precise coregistration to MNI (or another stereotactic) space. This atlas complements existing *in vivo* neuroimaging atlases of other brain structures (Tzourio-Mazoyer et al., 2002; Desikan et al., 2006; Destrieux et al., 2010).

We generated labels for two (bilateral) mescencephalic tegmental nuclei ICl/r and SCl/r. They were hypointense oval shaped structures easily identifiable on a T_2_-weighted MRI. The periaqueductal gray and the cuneiform nucleus were located at the anteromedial border of the IC (with good contrast in FA maps) and the cerebrospinal fluid limited its lateral edge. For the SC, its inferior edge was clearly located near the most rostral part of the IC, yet its rostro-ventral edge —in proximity to the thalamus— was less clearly defined due to the absence of an abrupt change in contrast in that area. The periaqueductal gray neighbored the medial border of the SC, while the cerebrospinal fluid delimited the posterolateral border of this structure. As visible in Table 1, our IC volumes were within literature values (specifically, closer to the inferior range of the latter), mainly derived from histology and structural MRI studies. Instead, the volume of the SC was slightly lower than reported literature values. Further validation work with MRI and histology might better elucidate the relationship between the achieved contrast in *in vivo* T_2_-weighted images and the distribution of iron (or of other microstructural properties) within different layers of this nucleus (Drayer et al., 1986).

For the manual delineation of the thalamic auditory MG nucleus we mainly used the T_2_-weighted image contrast. The MG was visible as a hypointense area neighboring (at its upper edge) the posteroventral thalamic nuclei, bounded inferiorly by the cerebrospinal fluid and limited by the pulvinar on its posterolateral border. Its medial border was clearly visible in FA maps, due to the high contrast with neighboring white matter bundles, such as the medial lemniscus and the spinothalamic tract. It is interesting to note the high variability in the MG volume values reported in the literature (see Table 1) for this structure. For instance, fMRI studies reported larger MG volumes compared to histology and structural MRI studies, possibly due to partial volume effects and artifacts due to draining veins in this region (Sitek et al., 2019). The MG volumes obtained in this study were within the range of previous literature reports (see Table 1). The MG label was useful to delimitate the posteromedial border of the LG, yet for the other borders of the LG we also used the contrast available in the FA maps. Notably, we used the FA maps to identify white matter bundles neighboring the LG, such as the optic tract anterior to LG and, in its medial aspect, the mesencephalic peduncles. The LG has an array of six cell layers currently not visible in our MRIs. Interestingly, recent *in vivo* studies report changes in the volume and shape of LG compatible with the location of the magnocellular layer in dyslexia according to previous hypothesis for this disease (Giraldo-Chica et al., 2015; Giraldo-Chica and Schneider, 2018). Previous reports of the LG volume show high degree of variability (up to two-fold) between individuals even when using the same techniques (Andrews et al., 1997; Selemon and Begovic, 2007; Kitajima et al., 2015; Wang et al., 2015). The LG volumes obtained in the present study were within the range of literature values (see Table 1). In the current study we found that the right LG was slightly larger compared to the left LG, in agreement with previous reports (Li et al., 2012; Lee et al., 2014).

The SOC was a round-oval hypointense area in FA maps with well-defined borders, yet at its most postero-lateral aspects its boundaries were more difficult to delineate due to its close proximity with other structures (e.g., the facial nucleus) (Kulesza, 2007). Our probabilistic SOC label is compatible with a complex comprising five nuclei described in the Paxinos atlas: the medial and lateral superior olive, the lateroventral and medioventral periolivary nucleus and the superior paraolivary nucleus (Paxinos et al., 2012). Previous histological and structural MRI studies report smaller SOC volumes (less than 6 mm^3^) than in the current work, most probably because they constrain their investigation to the small medial and lateral superior olivary nuclei (Glendenning and Masterton, 1998; Hilbig et al., 2009), and exclude the larger periolivary nuclei as well as the superior paraolivary nucleus. Yet, fMRI results report a larger volume (124 mm^3^) of the SOC, and our findings are within the range of literature values.

The atlas was validated with measures of inter-rater agreement and internal consistency of label delineations evaluated with the modified Hausdorff distance, a metric mostly unbiased by the size of the nucleus. We also evaluated the inter-rater agreement and the internal consistency using the Dice similarity coefficient, a more commonly used index in the neuroimaging literature for larger cortical and subcortical structures (Kaur et al., 2014; Iglesias et al., 2016; Tona et al., 2017). However, its sensitivity (similarly, to the Jaccard index) is biased toward larger structures with poorer performance in smaller structures such as tiny brainstem nuclei.

### Potential Impact of the Atlas

The current work presents a human *in vivo* probabilistic atlas of auditory and visual subcortical nuclei in stereotactic (e.g., MNI) space.

Upon its release on public repositories of neuroimaging data and tools, this atlas might be used as a tool to better identify the IC, SC, MG, LG, SOC nuclei location in conventional (e.g., 3 Tesla) MRI, and enable more accurate characterizations of auditory and visual connectivity pathways in fMRI and diffusion tractography studies. The current work provides a detailed description of the location, shape and anatomy of these nuclei that could be useful in research as well as in future clinical studies to aid treatment delivery and tuning of strategies such as repetitive transcranial magnetic stimulation, neurosurgical planning or focused ultrasound therapies i.e., in tinnitus treatment (Smit et al., 2016; Yilmaz and Yilmaz, 2018) or advanced stages of Parkinson’s disease with catalepsy symptoms (Engelhardt et al., 2018). This atlas could also be used to evaluate microstructural changes in the brainstem, such as the location of microbleeds/lesions/tumors.

We foresee that this atlas might improve the reconstruction of three dimensional auditory/visual tracts by providing more precise seeding information in diffusion tractography using MRI. Further, this tool might be applied to improve diagnosis, prognosis and therapeutic decisions for different pathologies, such as glaucoma, amblyopia, optic neuritis, hemianopsia, congenital or genetic abnormalities that involve alterations in auditory or visual pathways (i.e., congenital blindness or congenital deafness), macular degeneration, cognitive (i.e., dyslexia) and psychiatric disorders (i.e., schizophrenia) among other applications (Brown et al., 2016; Prins et al., 2016).

### Limitations and Future Perspectives

In the delimitation of LG and SOC we mainly used the contrast of FA maps, assuming that the observed low FA values within these nuclei were due to low myelin content (e.g., as expected in a gray matter region). To further corroborate our findings future human postmortem brain imaging with histological validation is needed to rule out possible confounds (such as low diffusion anisotropy due to crossing fibers). High angular resolution diffusion imaging (HARDI) acquisitions (preferentially with *b*-values > 2000 s/mm^2^) coupled with inspection of fiber orientation distributions and the application of tractography methods (Tournier et al., 2004; Zhang et al., 2012; Dell’Acqua and Tournier, 2019) could also provide useful information for brainstem nuclei delineation (such as a more accurate identification of neighboring fibers and of fibers traversing a nucleus, yet with higher uncertainty at the level of crossing fibers). At ultra-high magnetic fields, shorter *T*_2_ values might reduce the signal to noise ratio of high *b*-value diffusion images; yet, this effect is expected to be counterbalanced by gains in sensitivity due to the increased field strength.

We created an atlas based on images of healthy young adults, yet future work needs to be done in order to acknowledge possible differences across the lifespan. Future work should also investigate gender differences in nuclei shape and location.

Despite these limitations, our subcortical atlas is a unique non-invasive *in vivo* morphological tool to study the anatomy of deep brainstem and thalamic nuclei in healthy subjects, and its possible changes in different clinical populations.

## Conclusion

We foresee the use of the generated probabilistic atlas of the IC, SC, MG, LG and SOC to aid the localization of these nuclei in conventional (e.g., 3 Tesla) images in future research studies of auditory and visual functions. Further, this atlas, upon coregistration to clinical MRI, might improve the evaluation of lesions and the assessment of connectivity pathways underlying auditory and visual mechanisms in a broad set of disease populations (e.g., auditory agnosia, pure-word deafness, eye movement and visual field deficits, Parkinson’s hallucinations, and glaucoma).

## Data Availability

The datasets for this manuscript are not publicly available because we are using this data-set to currently expand our atlas and connectome of brainstem nuclei, as promised to a federal funding agency. The data-set, atlas and connectome will be released upon termination of this work. Requests to access the datasets should be directed to MB, martab@mgh.harvard.edu.

## Ethics Statement

Twelve healthy subjects (6m/6f, age 28 ± 1 years) provided informed and written consent for 7 Tesla MRI (Magnetom, Siemens Healthineers, Germany) per Massachusetts General Hospital Institutional Review Board approval in accordance with the Declaration of Helsinki.

## Author Contributions

NT and MB preprocessed and analyzed the data. MG-G, CS, and MB manually labeled the ROIs. MG-G, CS, KS, and MB wrote the manuscript. MB designed the research, performed the experiments, and secured the funding. BR and LW gave feedback along the process.

## Conflict of Interest Statement

The authors declare that the research was conducted in the absence of any commercial or financial relationships that could be construed as a potential conflict of interest.
